# Idiopathic giant abdominal lymph cyst: a case report

**DOI:** 10.1186/1752-1947-5-21

**Published:** 2011-01-19

**Authors:** Thorsten H Ecke, Holger Gerullis, Christoph J Heuck, Steffen Hallmann, Carsten Lange, Jürgen Ruttloff

**Affiliations:** 1 HELIOS Hospital, Department of Urology, Bad Saarow, Germany; 2 Lukas Hospital, Department of Urology, Neuss, Germany; 3 West German Cancer Center, University of Essen, Essen, Germany; 4 Montefiore Medical Center, Department of Medical Oncology, The Albert Einstein Cancer Center, New York, USA

## Abstract

**Introduction:**

Giant lymph cysts are a relatively frequent complication after surgical procedures in the abdomen, often after kidney transplantation, but there are also cases after pelvic surgery such as lymphadenectomy and others. In the recent literature, there have been no reported cases of idiopathic giant lymphocyst.

**Case presentation:**

We present the case of a 76-year-old Caucasian man who had a lymph cyst he had known of for more than 15 years. Laparoscopic treatment was necessary because of hydronephrosis of the left kidney.

**Conclusion:**

This case shows that laparoscopic drainage and partial resection of the lymph cyst is a safe and effective treatment.

## Introduction

Lymphoceles are bothersome postoperative complications, most frequently occurring after pelvic or retroperitoneal lymphadenectomy or renal transplant surgery [1]. Symptomatic, obstructive and infectious complications contribute to the morbidity caused by lymphoceles [2]. The diagnosis of lymphocele is made in the appropriate clinical setting, although lymphocele can occasionally be confused with urinoma, seroma, hematoma or abscess.

The method of treatment is controversial, ranging from conservative observation to aggressive deperitonealizing marsupialization at surgery [3-5].

This report concerns a very rare case of an idiopathic giant lymph cyst and its laparoscopic treatment.

### Case presentation

A 76-year-old Caucasian man was referred to our Department of Urology with a low-risk urothelial bladder cancer. The patient told us about an abdominal lymph cyst that he had known about for more than 15 years; a puncture revealed no malignant cells. His surgical history consisted of an open cholecystectomy twenty years ago. Twenty months after first presentation with bladder cancer, ultrasound examination and a computed tomography (CT) scan showed a giant lymph cyst. In the past years, the patient noticed a gradual increase in abdominal girth. Physical examination revealed a well-nourished man (height, 1.79 m; weight, 94.0 kg) with a grossly distended abdomen without ascitic fluid wave or tympany.

**Figure 1:**
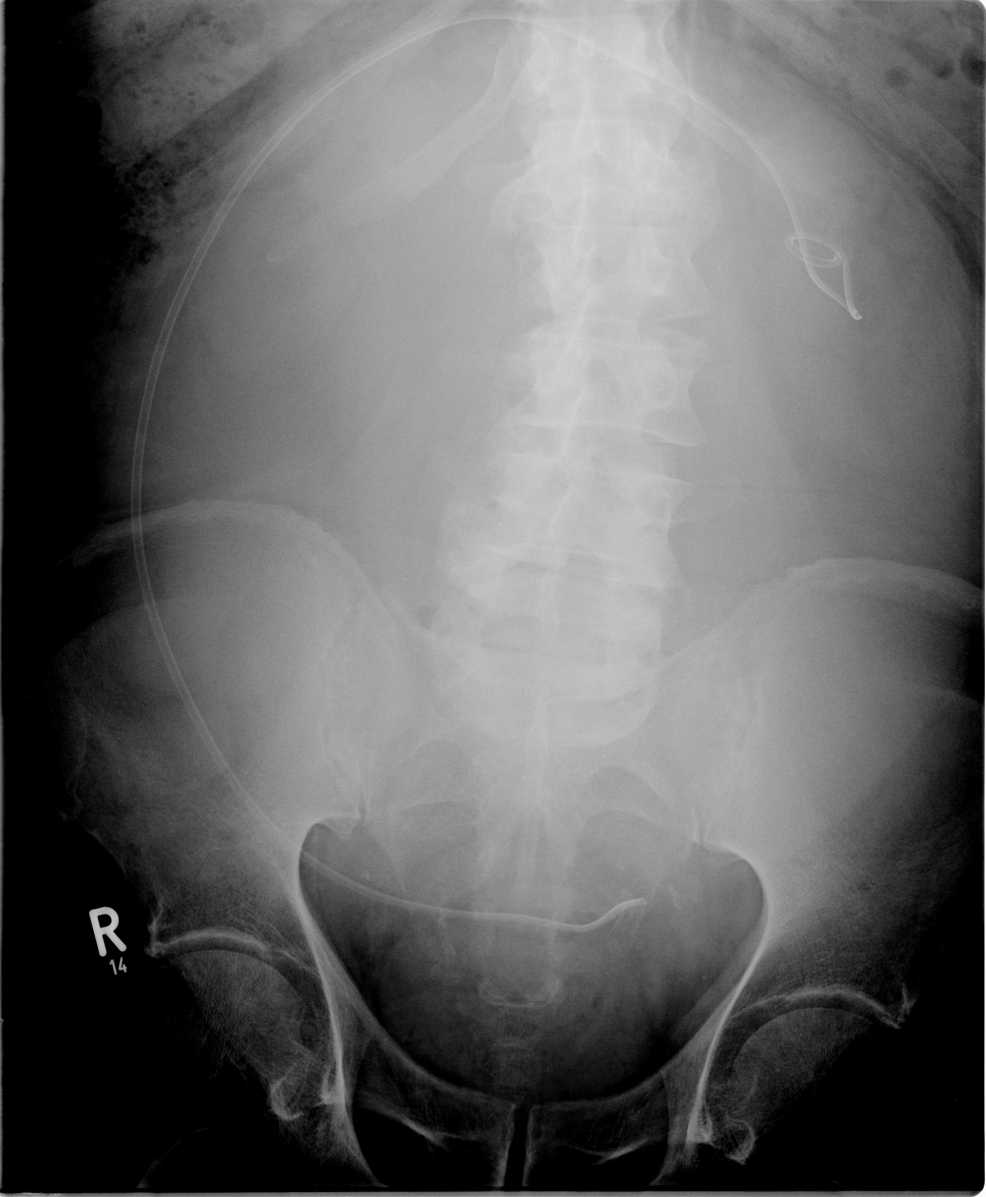
**Radiograph after ureter stent insertion**.

**Figure 2:**
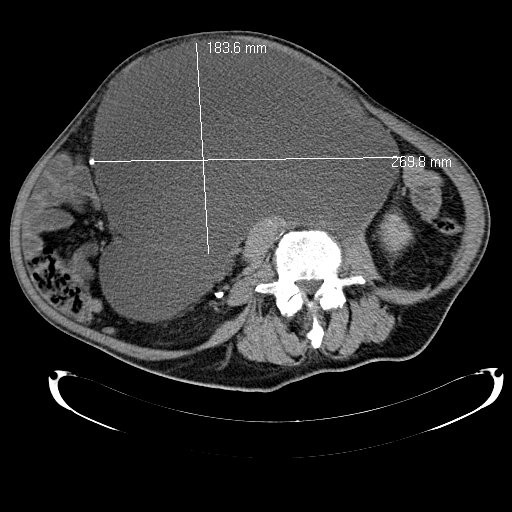
**Computed tomography scan of giant lymph cyst**.

**Figure 3:**
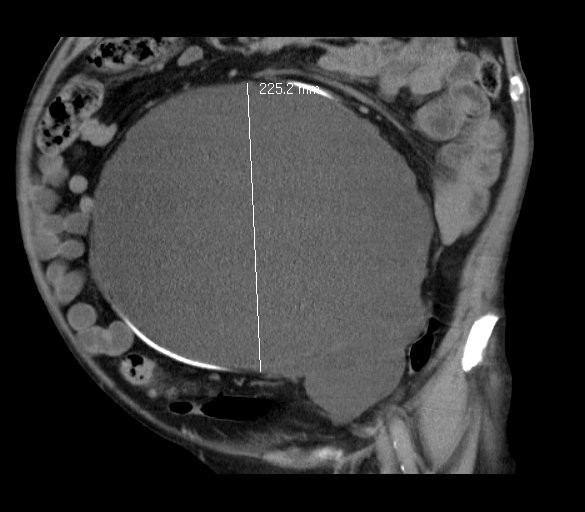
**Computed tomography scan of giant lymph cyst**.

Eleven months later, grade II hydronephrosis caused by the giant lymph cyst was visible in the patient's ultrasound examination. The kidney function test (clearance) showed a less-than-normal tubular function of 139 mL/min/1.73 m^2 ^body surface (lowest norm, 143 mL/min/1.73 m^2 ^body surface), left kidney with 27% of function and hydronephrosis. The serum creatinine level was 105 μmol/L. At that time, he had the first symptoms because of this giant lymph cyst; an insertion of a 70-cm-long ureteral catheter on the left side was possible (Figure 1). Postoperative ultrasound examination showed a complete regredience of hydronephrosis. Figures 2 and 3 show the giant lymph cyst (27 × 18.5 × 22.5 cm) in CT scans one month later.

Time delay of further treatment was caused by a new diagnosed atrial fibrillation. After cardiologic diagnostics and medical treatment two months later, laparoscopic partial resection of the giant lymph cyst was performed. Percutaneous drainage of a portion of the fluid was necessary to create adequate space for port placement. After drainage of one litre of fluid, a right-sided midabdomen 10-mm trocar was achieved, a second 5-mm trocar was inserted in the left side. In total, the mechanical aspirated volume during the operation was eight litres. The color of the fluid was more tan or brown than the straw-colored fluid typical of seromas or dark red of hematomas. Microscopic evaluation of the fluid revealed mostly lymphocytes and red blood cells; in the resected wall of the lymph cyst, collagen and muscle fiber were found, with no malignancy. Laboratory study was consisted with lymphatic fluid as well; the result was sterile lymphatic fluid. After surgery, a prolonged secretion of lymph fluid for more than 10 days was obvious. External pressure of the abdomen was necessary.

**Figure 4:**
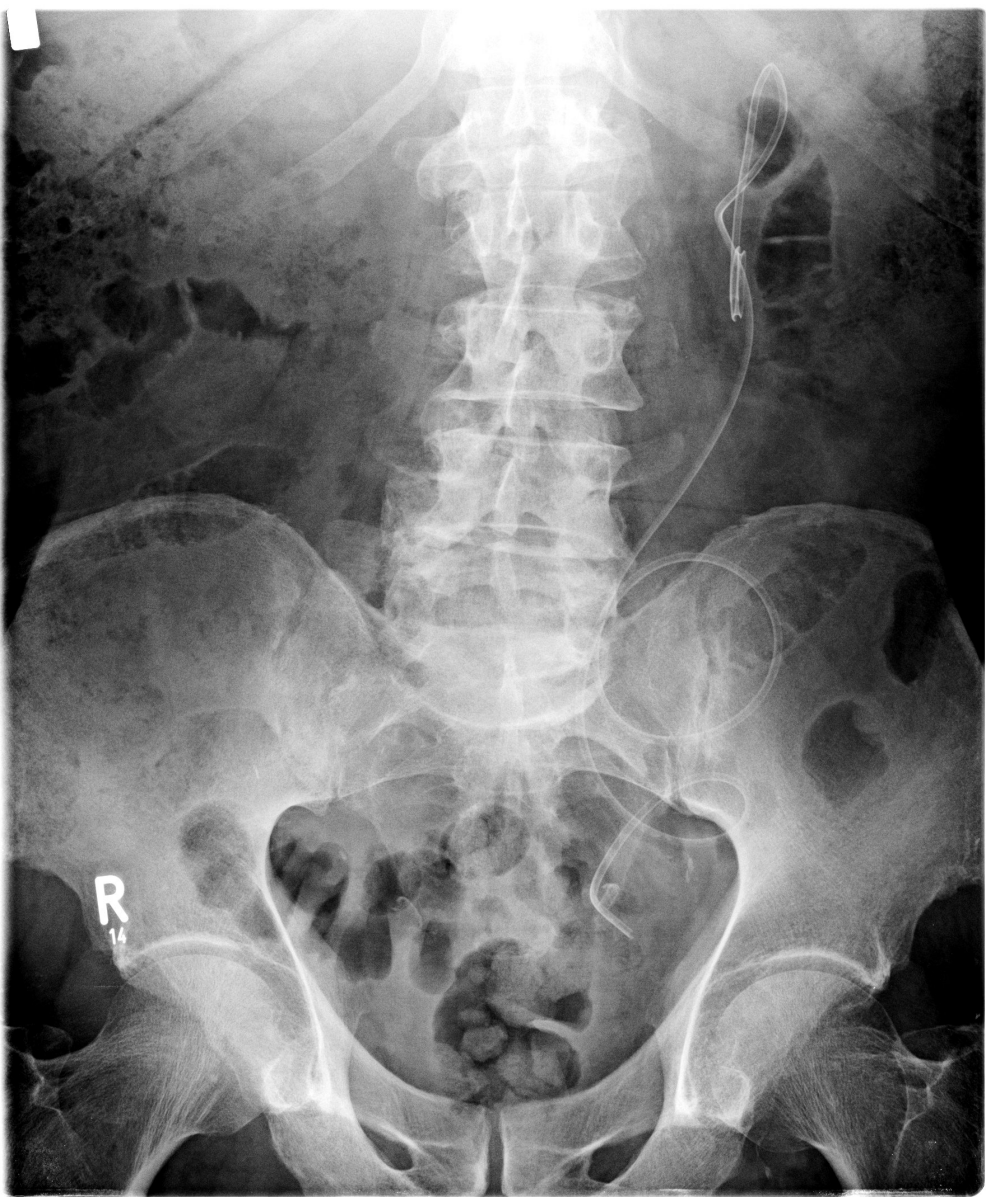
**Radiograph after laparoscopic operation**.

Two months later, the lymph secretion interrupted. The position of the elongated left ureter is shown in Figure 4. After extraction of the ureteral catheter by ureteroscopy, the kidney function test (clearance) again showed a less-than-normal tubular function of 137 mL/min/1.73 m^2 ^body surface (lowest norm, 143 mL/min/1.73 m^2 ^body surface), and the left kidney was now better with 39% of function and better outlet. The serum creatinine level was 92 μmol/L.

## Discussion

Pelvic and retroperitoneal lymphoceles may cause venous obstruction with subsequent edema and thromboembolic complications. Large lesions cause abdominal distension, and pain may become unbearable as the lymphoceles fill much of the abdomen [1,2,4,6].

Although lymphoceles are rarely fatal, they create a number of problems, some of which may be serious. They may compromise kidney function by various means: ureters may become obstructed, secondary infection may occur and blood supply may be diminished [1,2,4,6]. As shown in this case report, obstruction of a ureter and consecutive hydronephrosis is an important reason to treat the patient.

Pelvic and retroperitoneal lymphoceles may cause venous obstruction with subsequent edema and thromboembolic complications. Large lesions cause abdominal distension, and pain may become unbearable as the lymphoceles fill much of the abdomen.

Lymphoceles may appear from days to years [4,6]; an interval of several weeks is typical. The mode of therapy for lymphoceles is a matter of controversy, reflecting the difficulty in management. Options have included observation, dietary restriction, blind needle aspiration, external drainage and marsupialization [1-6]. Size is prognostic: small collections frequently resorb spontaneously, but large collections are not as likely to resolve spontaneously and usually require treatment [4,7].

Ultrasound examination is excellent in the detection of lymphoceles. Lymphoceles generally occur in areas amenable to ultrasonography such as the pelvis, abdomen or retroperitoneum [1,6]. Ultrasound findings of hypoechoic to anechoic masses with through transmission, occasionally with septa and dependent or scattered debris, are suggestive but not specific for the diagnosis. Lymphoceles with substantial debris and internal echoes are more likely to be infected [1,7].

CT displays characteristic, albeit not pathognomonic, signs. When uncomplicated, lymphocele masses produce low attenuation values. Negative CT numbers usually indicate fat content and are highly suggestive of lymphocele. Low CT numbers could effectively give an indication for abscess and hematoma from consideration and are helpful diagnostically. Thoracic and idiopathic lymphoceles are relatively uncommon [1].

Diagnosis by needle aspiration is fairly straightforward and includes gross inspection and chemical examination of the fluid (to exclude urinoma) and Gram stain and cytology to exclude infection and tumor. The appearance of the lymphocele fluid varies from tan to dark yellow or brown, depending on the amount of fat.

Because most lymphoceles resolve spontaneously, drainage should be reserved for large or symptomatic collections. Treatment options for symptomatic lymphoceles include percutaneous, open and laparoscopic drainage. Although performed with minimal difficulty, percutaneous drainage has a 50% to 80% rate of recurrence and therefore is often reserved for diagnosis [1,8]. Catheter drainages that successfully treated the lymphoceles were relatively long term. In most reports, curative catheter drainage lasted up to five weeks or an average of 18 days [1-4]. CT was the predominant guide for drainage because it permits precise localization of the position of the collection and of the adjacent ureter and bowel [1].

The open drainage procedure provides definitive therapy for symptomatic lymphoceles; however, patients experience discomfort because of the laparotomy incision and may remain hospitalized for five to seven days [8].

In 1991, McCullough *et al. *[9] described the first successful laparoscopic drainage of a lymphocele. Lymphocele recurrence is seen in 7% to 25% of laparoscopically drained lesions [10,11]. In 1992, Gruessner *et al*. [10] reported a 64% success rate with the laparoscopic approach to anterior or superior lymphoceles with no complications in a group of 14 patients. Their analysis suggested that the development of the laparoscopic method of draining lymphoceles has reduced both the morbidity and the overall cost of care for transplant patients. With the assistance of ultrasound examination, the risk of injury can be minimized. Nowadays the laparoscopic management should be the first-line treatment of lymphoceles [12].

The size of this giant lymph cyst created a technical challenge. Minimizing morbidity remained a priority; the laparoscopic approach was attempted for this reason. Three ports were needed for the identification, manipulation and stapling of the cyst wall. In this particular case, the cyst wall was easily seen. If difficulty occurs, either laparoscopic or external ultrasound examination could be used to ascertain the location of any other cyst. Furthermore, the placement of a pigtail catheter externally into the cyst or methylene blue injection into the lymphocele could facilitate laparoscopic viewing [13]. In our case, after CT scan and ultrasound examination, there was only a single giant lymph cyst.

Reports of giant lymphoceles in either the immediate or distant posttransplant period are sparse. Most patients present earlier in the development of a lymphocele with complaints that include decreased renal function or increased creatinine, perirenal discomfort, cutaneous fistula formation or weight gain. This patient did not have any complaints; increased abdominal girth, hydronephrosis in ultrasound examination and decreased renal function occurred very late. Because the patient was symptomatic on presentation, we considered surgical drainage the best therapeutic option. The laparoscopic approach was used initially in the attempt to minimize the patient's discomfort and hospital stay. To prevent reaccumulation of the lymph, a large peritoneal window was created. Despite the large size of this lymphocele and the extent of the laparoscopic dissection required, the patient was able to be discharged from the hospital 13 days after the procedure without any untoward events.

## Conclusion

This case provides evidence that laparoscopic drainage should not be restricted to small and uncomplicated lymphoceles. Complex and giant lymphoceles may be drained safely and effectively by laparoscopy as well.

## Competing interests

The authors declare that they have no competing interests.

## Consent

Written informed consent was obtained from the patient for publication of this case report and accompanying images. A copy of the written consent is available for review by the Editor-in-Chief of this journal.

## Authors' contributions

THE was involved in drafting the manuscript and in the review of the literature and in performing the clinical follow-up. HG was involved in drafting the manuscript and in the review of the literature. CJH was involved in drafting the manuscript and in the review of the literature. SH participated in the surgery and was involved in the clinical follow-up. CL was involved in the review of the literature and the clinical follow-up. JR was involved in the clinical follow-up and supervised this report. All authors read and approved the final draft.
